# Evaluation of the Ronnie Gardiner Method in individuals with stroke in the late phase of recovery: a protocol for a single-blind multicentre randomised controlled trial

**DOI:** 10.1136/bmjopen-2025-107178

**Published:** 2026-02-04

**Authors:** Shashank Ghai, Petra Pohl

**Affiliations:** 1 Department of Political, Historical, Religious and Cultural Studies, Karlstad University, Karlstad, Sweden; 2 Centre for Societal Risk Research, Karlstad University, Karlstad, Sweden; 3 Department of Health and Rehabilitation, Institute of Neuroscience and Physiology, University of Gothenburg Sahlgrenska Academy, Gothenburg, Sweden

**Keywords:** Stroke, REHABILITATION MEDICINE, Gait, Cognition

## Abstract

**Background:**

Stroke is a prevalent neurological condition that frequently results in long-term disabilities and considerable societal costs. While existing rehabilitation approaches provide some benefits, residual motor impairments often persist and become permanent, leading to ongoing activity restrictions. Music-based intervention, such as the Ronnie Gardiner Method (RGM), adheres to best practice principles of stroke rehabilitation by simultaneously engaging motor, sensory, cognitive and emotional functions, potentially offering enhanced recovery outcomes. However, research examining its effectiveness in chronic stroke rehabilitation remains limited.

**Methods and analysis:**

This multicentre, randomised controlled trial will recruit 84 community-dwelling individuals with chronic stroke over a 2-year period across four sites in Sweden. Participants will be randomly assigned to either an intervention group receiving RGM training (n=42) or a passive waitlist control group (n=42). Only the investigators and outcome assessors will remain blinded to group allocation. RGM training consists of 60 min group sessions twice weekly for 12 consecutive weeks. The primary outcome is to evaluate the effectiveness of RGM training on balance performance using the Mini-Balance Evaluation Systems Test. Secondary outcomes include assessment of gait function (10-Metre Walk Test, 6 min Walk Test, Short Physical Performance Battery), upper limb function (9-Hole Peg Test, Observational Drinking Task Assessment), cognitive abilities (Victoria Stroop Test, Rey Complex Figure Test, Memory Test), fear of falling (Falls Efficacy Scale-International) and stroke-related functional impact (Stroke Impact Scale-16). Broader health-related quality of life will be assessed using the RAND 36-Item Health Survey, EuroQol 5-Dimension 5-Level, and depressive symptoms will be measured with the Montgomery-Åsberg Depression Rating Scale. All outcomes will be assessed at baseline, postintervention and at a 3-month follow-up. Additional assessments will include qualitative evaluations of participants’ and trainers’ subjective experiences, cognitive screening (Montreal Cognitive Assessment) and postintervention enjoyment assessment (Physical Activity Enjoyment Scale).

**Ethics and dissemination:**

Ethical approvals for the study have been obtained from the Swedish Ethical Review Authority (Dnr: 2025-01269-01 and Dnr: 2025-08232-02). The results will be disseminated via peer-reviewed journal publications, conference presentations and targeted communication with stakeholders and the media.

**Trial registration number:**

NCT06979050.

**STRENGTHS AND LIMITATIONS OF THIS STUDY**

- This work reports on a protocol for multicentre, single-blind randomised controlled design which will evaluate the effects of a music-based intervention among chronic stroke survivors.
- The study incorporates quantitative assessments that comprehensively evaluate measures spanning balance, motor function, cognition and quality of life, whereas qualitative assessments evaluate experiences of participants and trainers delivering the intervention.
- A limitation of the work is that complete blinding of the participants and the trainers is unfeasible due to the nature of the intervention.

## Introduction

 Stroke remains one of the leading causes of disability worldwide, affecting approximately 12 million people annually and resulting in significant long-term impairments that impact quality of life and impose substantial economic burdens on healthcare systems and society.^1 2^ The majority of stroke survivors experience persistent motor, cognitive and sensory deficits that limit their ability to perform activities of daily living and participate fully in community life.^3 4^ Balance impairment is particularly prevalent among stroke survivors, with studies indicating that up to 40%–70% of individuals experience balance problems during the chronic phase of recovery,^5 6^ leading to increased fall risk, reduced mobility and decreased independence.^7^ These balance deficits not only affect physical function but also contribute to reduced confidence in movement, social isolation and decreased overall well-being.^8 9^

Current stroke rehabilitation approaches, while beneficial, often yield modest improvements in functional outcomes, particularly in the chronic phase of recovery when neuroplasticity is thought to be reduced.^10^,^13^ Traditional physiotherapy interventions focusing on repetitive task-specific training have shown effectiveness but may lack the engagement and motivation necessary for sustained participation in long-term rehabilitation programmes.^14 15^ The complex nature of stroke-related impairments suggests that interventions targeting multiple domains simultaneously may be more effective than approaches focusing on isolated functions.^16^ This understanding has led to increased interest in multimodal rehabilitation strategies that can simultaneously address motor, cognitive, sensory and emotional aspects of recovery.^17^

Music-based interventions have emerged as a promising approach in neurological rehabilitation, leveraging the brain’s inherent capacity for neuroplasticity through multisensory stimulation and engagement.^18 19^ The neurobiological basis for music therapy lies in the widespread activation of neural networks during musical activities, including areas responsible for motor control, sensory processing, cognitive function and emotional regulation.^18^ Research has demonstrated that musical activities can facilitate neuroplastic changes, promote motor learning and enhance cognitive function in stroke.^20^,^22^ Specifically, rhythmic entrainment, driven by the rhythmic components of music, has been thought to provide external timing cues that can synchronise motor output and enhance coordination in both gait and upper extremity movements, particularly in individuals with stroke-induced neurological impairments.^23^,^27^

The Ronnie Gardiner Method (RGM) represents a structured approach to music-based rehabilitation that combines rhythmic music with coordinated movements, cognitive challenges and social interaction.^28 29^ This method, developed by jazz musician and drummer Ronnie Gardiner, involves participants performing specific bilateral activities from upper and lower extremities while following musical rhythms, requiring simultaneous coordination of motor, cognitive and sensory functions.^29^ RGM is primarily considered as a complementary approach with traditional rehabilitation methods and offers a variety of person-centric, challenging activities for individuals with different inherent functional capacities, using a mix of symbols, colours, sounds and sensory stimulations.^29^ The approach strongly adheres to established best practice guidelines in stroke rehabilitation, including key principles such as high repetition, task specificity, individualised therapy, progressive difficulty and multimodal sensory input.^30 31^ Furthermore, the group-based format of RGM sessions also promotes social interaction and peer support,^29^ which may additionally enhance motivation, adherence and psychological well-being while maintaining effectiveness comparable to individualised therapy.^32 33^

Preliminary evidence suggests that RGM may be particularly beneficial for balance and motor function in neurological populations such as those with Parkinson’s disease,^34 35^ though research specifically examining its effectiveness in chronic stroke rehabilitation remains limited.^36^,^38^ The potential for RGM to simultaneously target multiple impairment domains while providing an engaging and enjoyable therapeutic experience makes it a valuable addition to rehabilitation, especially for stroke survivors who may be experiencing reduced motivation or plateau in their recovery.^39^ Given the challenges associated with traditional approaches to chronic stroke rehabilitation and the promising theoretical foundation of music-based interventions, there is a compelling need for rigorous multifaceted evaluation of RGM effectiveness in this population. Therefore, this study aims to investigate the effectiveness of RGM training on balance performance, related functional outcomes, cognitive abilities, quality of life and intervention-related costs with RGM in community-dwelling individuals with chronic stroke through a randomised controlled trial design.

### Study objectives

The primary objective of this study is to evaluate the effectiveness of the RGM, a rhythm-based and music-based training programme, on improving balance in individuals who are in the chronic phase of stroke recovery, defined as more than 6 months poststroke. The Mini-Balance Evaluation Systems Test (Mini-BESTest) will be used as the primary outcome measure to assess changes in balance and functional mobility. The secondary objectives are to examine the effects of RGM on other key domains of poststroke rehabilitation, including gait, upper limb function, cognitive abilities such as working memory and divided attention, stroke-related functional impact and health-related quality of life. In addition to these quantitative measures, the study also aims to explore qualitative aspects of the intervention by investigating participants’ and trainers’ experiences related to motivation, engagement and perceived impact on daily participation. These objectives together aim to provide a comprehensive understanding of the clinical utility and potential implementation of RGM as a component of community-based stroke rehabilitation.

## Methods and analysis

### Study design

This study will follow a single-blind, multicentre, mixed-method, randomised controlled trial design with a parallel-group structure and a 1:1 allocation ratio. Blinding will be implemented for investigators responsible for outcome assessments and data analysis, who will remain unaware of group allocation throughout the study period. Due to the nature of the intervention, complete blinding of participants and intervention providers (certified trainers) is not feasible. Online supplemental file 1 contains a completed Standard Protocol Items: Recommendations for Interventional Trials (SPIRIT) outcomes 2025 checklist,^40^ while online supplemental file 2 provides registration information for the trial and details about the trial sponsor.

### Participants

Participants will be eligible for inclusion if they meet all of the following criteria:

1. Community-dwelling stroke survivors aged 18 years or older.
2. Stroke that occurred more than 6 months prior to enrolment.
3. Adequate cognitive function as demonstrated by a Montreal Cognitive Assessment (MoCA) score of 25 or higher out of 30 points.
4. Mild to moderate functional disability corresponding to a Modified Rankin Scale (mRS) score of 1–3.
5. Sufficient independence to participate without assistance in daily activities required for study participation, including the ability to independently travel to measurement and training sites and use restroom facilities.
6. Adequate mobility is defined as the ability to stand unsupported for 2 min and walk 10 m with or without assistive devices but without supervision from another person.

Participants will be excluded if they have any of the following:

1. Severe visual and/or hearing impairments that would significantly interfere with participation in the study protocol.
2. Participation in regular sessions with the RGM within the past 1 year, that is, after August 2024.
3. Previous experience playing a musical instrument, defined as practicing more than 1 hour per week within the past 10 years.

### Intervention

The RGM is a comprehensive neuroplasticity-based intervention that combines multisensory stimulation through rhythm, music, colour, speech, text, shapes and coordinated movements to enhance cognitive and motor functioning in individuals with neurological conditions. This evidence-based programme is implemented across Swedish rehabilitation and healthcare centres and will be adapted for the current study population.

The intervention will use a unique symbol system comprising four distinctive red and blue symbols resembling hands and feet that will be projected on a screen within specialised note systems called ‘choreoscores’.^35^ The colour-coding will represent lateralised brain activity, with red symbols corresponding to right hemispheric control of left-sided body movements and blue symbols representing left hemispheric control of right-sided movements.^41^ This system creates 19 possible symbol combinations; each associated with specific movements and four-letter verbal cues imitating sounds inspired from drumming (such as ‘BOOM,’ ‘CHIC,’ ‘TING’) that participants will pronounce loudly while executing corresponding motor actions to rhythmical music. The multitasking nature of RGM will require participants to simultaneously process visual symbols, execute corresponding movements, vocalise specific verbal cues, maintain balance, follow musical rhythm and attend to practitioner instructions. This complexity is designed to improve dual-task performance and movement automaticity while promoting neuroplastic adaptations through repeated practice of cognitively demanding motor tasks.

The RGM protocol will incorporate multiple external cueing modalities including visual cues (screen-projected symbols), auditory cues (beat-based popular music) and somatosensory cues (body percussion through handclaps, foot stamping and thigh slapping). Music selection will be individualised, when possible, to incorporate participants’ preferences, with tempo measured in beats per minute to enable objective progression monitoring. The intervention will be delivered in a standing position to enhance postural stability through weight shifts that activate anticipatory and reactive postural control mechanisms, with seated adaptations available, when necessary, based on individual participant needs.

Training sessions will be led by a certified RGM practitioner. Each 60 min group session will follow a structured format beginning with a 5 min warm-up period incorporating light stretching, relaxation techniques and breathing exercises. This will be followed by systematic progression through the symbol-based activities starting at a slow tempo of 50 beats per minute and gradually increasing in complexity and pace according to individual participant capabilities. Sessions will conclude with a 5 min relaxation period and opportunities for social interaction among participants.

The complete intervention protocol will involve twice-weekly group sessions conducted over 12 consecutive weeks under the guidance of the certified RGM practitioner who will adjust activity complexity based on participants’ physical capacities. To ensure transparency and reproducibility, comprehensive implementation details will be reported in the final publication following the Robb *et al*^42^ checklist for reporting music-based interventions as recommended by Consolidated Standards of Reporting Trials (CONSORT) guidelines. This will include detailed information on trainer qualifications and experience levels, group size, specific music selections with tempo progressions, music delivery method, intervention setting and treatment fidelity.

### Patient and public involvement

The training frequency of RGM was determined through a collaborative ‘patient involvement’ process that involved potential participants, that is, community-dwelling individuals with stroke.^43^ These individuals were actively engaged in the planning phase to ensure their perspectives and preferences informed the intervention design, thereby enhancing participant engagement and adherence.^44^ Through this engagement, the research team learnt that the stroke survivors had concerns about time commitment when considering the initially proposed three sessions per week. Based on this feedback, a mutual decision was made to conduct sessions twice weekly rather than three times per week.

### Waitlist control group

Participants randomised to the waitlist control group will continue with their usual daily activities during the 12-week intervention period. They will not receive any study-specific interventions during this time but will complete all outcome assessments according to the same schedule as the intervention group. Following completion of the 3-month follow-up assessments, waitlist control participants will be offered the opportunity to participate in the RGM training programme.

### Outcomes

The periods when the outcome measures would be assessed are illustrated in table 1.

**Table 1:** Schedule of outcome assessments for the Ronnie Gardiner Method intervention.

Outcome measures	Baseline (Week 0)	Post-intervention (Week 12)	Follow-up (Week 24)
Primary outcome measure			
Mini-BESTest	x	x	x
Secondary outcome measures			
FES-I	x	x	x
Memory test (Immediate & Delayed Recall)	x	x	x
Victoria Stroop Test	x	x	x
Rev Complex Figure Test	x	x	x
SPPB	x	x	x
10MWT	x	x	x
6MWT	x	x	x
ODTA	x	x	x
9HPT	x	x	x
SIS-16	x	x	x
MADRS	x	x	x
RAND-36	x	x	x
EQ-5D-5L	x	x	x
Other pre-specified outcome measures			
MoCA	x		
PACES		x	
Focus Group Interviews		x	
Intervention-related costs	x	x	x

EQ-5D-5L, EuroQol 5-Dimension 5-Level; FES-I, Falls Efficacy Scale–International; 9HPT, 9-Hole Peg Test; MADRS, Montgomery–Åsberg Depression Rating Scale; Mini, BESTest, Mini–Balance Evaluation Systems Test; MoCA, Montreal Cognitive Assessment; 6MWT, 6-Minute Walk Test; 10MWT, 10-Meter Walk Test; ODTA, Observational Drinking Task Assessment; PACES, Physical Activity Enjoyment Scale; SIS-16, Stroke Impact Scale–16; SPPB, Short Physical Performance Battery.

#### Primary outcome

1. Mini-Balance Evaluation Systems Test (Mini-BESTest): the Mini-BESTest is a shortened version of the Balance Evaluation Systems Test (BESTest), designed to assess balance and functional mobility, especially in individuals with neurological or musculoskeletal conditions.^45^ It focuses on balance deficits across four main systems: anticipatory postural adjustments (preparing and adjusting posture for movements), reactive postural control (responding to unexpected balance challenges), sensory orientation (using sensory inputs such as vision and proprioception for balance) and dynamic gait (stability and efficiency while walking, including maintaining balance during movement). The Mini-BESTest consists of 14 items, making it quicker to administer than the full BESTest, yet still comprehensive. Higher scores indicate better balance control.

#### Secondary outcomes

1. Falls Efficacy Scale-International (FES-I): the FES-I assesses an individual’s confidence in performing 16 daily activities, such as walking indoors, getting dressed or visiting friends without falling.^46^ It captures the level of concern about falling during these tasks, which can affect participation in daily life. A higher FES-I total score signifies greater fear of falling. It is widely used in older adults and those at risk of falls to identify concerns and guide interventions aimed at improving balance and reducing fall risk.
2. 10-Metre Walk Test (10MWT): the 10MWT measures self-selected gait speed by timing how long an individual takes to walk 10 m on a flat surface.^47^ It evaluates functional mobility, gait efficiency and balance during walking. Faster times (ie, higher speeds) indicate better walking ability. It is commonly used in neurological and musculoskeletal rehabilitation to monitor progress and predict community ambulation capacity.
3. 6 min Walk Test (6MWT): the 6MWT measures the distance an individual can walk over 6 min on a flat corridor at a self-paced speed.^47^ It assesses aerobic capacity, endurance and functional mobility. Greater distances reflect better cardiovascular and pulmonary function and overall endurance. The test is widely used to monitor progress in rehabilitation and to evaluate interventions targeting endurance and functional capacity.
4. 9-Hole Peg Test (9HPT): the 9HPT is a timed measure of finger dexterity and fine motor skills.^48^ Participants pick up nine pegs, one at a time, and place them into nine holes on a board, then remove them. The total time to complete the task for each hand is recorded. Faster completion times indicate better hand function. It is frequently used to quantify upper extremity impairment in stroke, multiple sclerosis and other neurological conditions.
5. Stroke Impact Scale-16 (SIS-16): the SIS-16 is a self-reported questionnaire comprised of 16 items that measure physical and functional performance after stroke, including strength, hand function, mobility and activities of daily living.^49^ Respondents rate their ability to perform tasks such as walking a block or carrying a heavy object. Higher scores indicate better perceived physical function and quality of life. It helps track recovery, progress and guide rehabilitation planning.
6. Short Physical Performance Battery (SPPB): the SPPB assesses lower extremity function and mobility through three subtests—standing balance (side-by-side, semi-tandem and tandem positions), gait speed (timed 4 m walk) and lower-limb strength (timed five repeated chair stands). Each subtest is scored from 0 to 4, and the sum (0–12) indicates overall performance.^50^ Lower scores predict higher risk of disability, falls and mortality in older adults.
7. Memory Test (Immediate and Delayed Recall): this assessment evaluates verbal memory by having participants listen to a short text, immediately recall as much as possible, and then, after a brief delay during which another task is administered, recall the text again.^51^ The immediate recall score reflects initial encoding and short-term memory, while the delayed recall score reflects consolidation and retrieval processes. It is used to track changes in memory performance over time.
8. Victoria Stroop Test: the Victoria Stroop Test measures cognitive flexibility, selective attention and response inhibition.^52^ During the test, participants are presented with colour words printed in incongruent ink colours (eg, the word ‘red’ printed in blue ink) and must name the ink colour rather than read the word. Performance is scored based on reaction time and number of errors. It is commonly used to assess executive function deficits, particularly in individuals with neurological impairments.
9. Rey Complex Figure Test: this neuropsychological test evaluates visuospatial constructional ability, visual memory, attention and executive planning. Participants copy a complex geometric figure; scoring considers the accuracy and placement of design elements, with scores ranging from 0 to 18. It helps detect impairments in visuospatial and memory functions often seen after brain injury or stroke.^53^
10. Montgomery-Åsberg Depression Rating Scale (MADRS): the MADRS is a clinician-administered tool consisting of 10 items that assess the severity of depressive symptoms, including mood, tension, sleep, appetite, concentration and suicidal thoughts.^54^ Each item is scored on a scale from 0 (absent) to 6 (severe), with higher total scores indicating more severe depression. It is commonly used in clinical settings to monitor changes in depressive symptoms over time.
11. RAND 36-Item Health Survey (RAND-36): the RAND-36 assesses health-related quality of life across eight domains—physical functioning, role limitations due to physical health, role limitations due to emotional health, energy/fatigue, emotional well-being, social functioning, pain and general health perceptions.^55^ Each domain is scored from 0 to 100, with higher scores indicating better perceived health status. It is widely used in clinical research to capture patient-reported outcomes across multiple health dimensions.
12. Observational Drinking Task Assessment: this test evaluates upper limb function, motor control and coordination by observing a simulated drinking task.^56^ Participants reach for a cup, grasp it, bring it to the mouth and set it back down. Observers rate movement smoothness, compensatory strategies and time taken. It is often employed in neurological rehabilitation to assess functional use of the affected arm and hand.
13. EuroQol 5-Dimension 5-Level (EQ-5D-5L): the EQ-5D-5L is a standardised instrument to measure health-related quality of life across five dimensions—mobility, self-care, usual activities, pain/discomfort and anxiety/depression.^57^ Each dimension has five levels of severity (no problems to extreme problems). Responses generate a descriptive health state and an index value. A higher index value indicates better overall health. It is used in clinical trials and health-economic evaluations to compare health outcomes across populations.

#### Other outcome measures

1. Montreal Cognitive Assessment (MoCA): the MoCA is a brief screening tool to detect mild cognitive impairment. It covers multiple cognitive domains, including short-term memory recall, visuospatial abilities, executive functions, attention, concentration, language and orientation.^58^ The test yields a total score out of 30, with a score of 26 or above generally considered normal. It is commonly used to screen for cognitive deficits in stroke and other neurological populations.
2. Physical Activity Enjoyment Scale (PACES): the PACES assesses enjoyment related to physical activity through statements such as ‘I enjoy it’ or ‘It’s no fun at all’, rated on a Likert scale.^59^ Higher total scores indicate greater enjoyment of exercise. It is used to understand motivation and predict adherence to physical activity programmes.

### Intervention-related cost evaluation

All direct intervention costs related to implementing the RGM across the four sites will be systematically captured and reported. These costs will include trainer fees, facility usage, equipment and materials, staff time for coordination and administration.

### Focus group interviews

Focus group interviews will be conducted to shed light on how participants and RGM trainers subjectively experience RGM training. Through these focus group interviews, the aim is to explore participants’ experiences of engaging with the RGM in a social setting, identify factors that influence motivation, engagement and adherence to the exercise programme, and generate insights on how RGM training could be further tailored to meet the needs of individuals living with stroke. Additionally, the interviews will highlight the trainers’ perspectives on adapting the tempo, music selection and exercises for this specific target group. Focus groups with participants and RGM trainers will be conducted separately and in person to ensure that both perspectives are captured in depth. An interview guide developed to structure these sessions is provided in online supplemental file 3.

### Recruitment procedure

Recruitment will take place via advertisements in Gothenburg, Karlstad, Stockholm, Malmö and through outreach to local stroke associations, which has already been completed. The advertisement will be shared on relevant Facebook pages for stroke survivors, including Stroke Mitt I Livet (‘stroke in midlife’), a targeted initiative within stroke associations across Sweden for individuals who experienced stroke during working age. Interested individuals will be instructed to email the responsible researcher (PP) to register their interest. Follow-up phone contact will be made with those who have emailed. The recruitment advertisement to be used is provided in online supplemental file 4.

**Figure 1:**
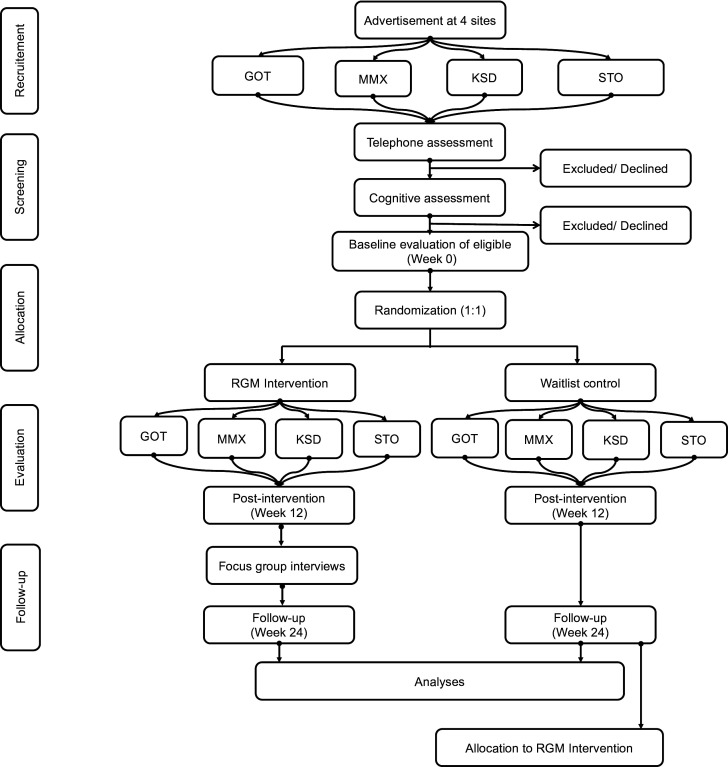
Schematics of the study procedure. GOT, Gothenburg; KSD, Karlstad; MMX, Malmö; RGM, Ronnie Gardiner Method; STO, Stockholm.

During the phone call, a structured script will be used to conduct a brief screening process. This includes administration of a telephone version of the mRS,^60^ and additional questions aligned with the study’s inclusion criteria. Individuals who appear eligible based on this initial screening will be invited to an in-person eligibility assessment in their respective city. At this appointment, a written informed consent would be sought by the responsible researcher (PP) after which cognitive screening will be conducted using the MoCA, with the resulting score determining final inclusion in the study. The participant information form and informed consent form to be used in the study are provided in onlinesupplemental files 5  6. Figure 1 provides an illustration of the study schematics.

### Randomisation procedure

Randomisation (1:1) will be conducted in four separate blocks corresponding to each study site: Gothenburg, Karlstad, Stockholm and Malmö. Microsoft Excel will be used for performing the randomisation based on participant identification numbers, with the process managed by an external researcher to ensure objectivity.

### Allocation concealment

An external researcher will be responsible for sending pre-prepared printed letters to both intervention and control groups, which will be placed in sealed envelopes to maintain allocation concealment. Outcome assessors (PP, SG) will remain blinded to group allocation throughout the study period. While complete blinding presents inherent challenges due to the nature of the intervention, several measures will be implemented to minimise assessment bias. Participants will receive explicit instructions not to disclose their group assignment during assessment visits, with these confidentiality instructions included in both written invitation letters and delivered verbally at each assessment session. Assessment protocols will be standardised to minimise opportunities for inadvertent unblinding, and any instances of unblinding will be documented and reported in the final analysis to maintain study integrity.

### Data management plan

Personal data gathered during the study will be pseudonymised immediately on inclusion and handled under strict confidentiality in accordance with Swedish data protection regulations and the University of Gothenburg’s Rules for Research Data Management.^61^ Data containing personal information will be stored on the Nextcloud files platform with regular automated backups and processed within the Trusted Research Environment platform. Paper-based assessment instruments will be archived in the local unit archive under restricted access protocols. All data and study-related documents will be preserved for a minimum 10-year retention period in compliance with institutional guidelines.^61^

Following study completion, anonymised data will be made available through the Swedish National Data Service (SND) repository under restricted access conditions, requiring individual approval for each data request. Interview recordings will not be published due to confidentiality requirements. Given the academic investigator-initiated nature of this study, no external data monitoring committee will be required. A detailed data management plan is provided in online supplemental file 7.

### Sample size calculation

The sample size estimation was conducted using GPower software (V.3.1.9.7). The study’s primary outcome is the between-group difference in change of Mini-BESTest total scores postintervention. Based on Beauchamp *et al*,^62^ a minimal clinically important difference of 4 points was chosen, with a corresponding baseline SD of 6.5 points for the Mini-BESTest in a stroke outpatient cohort. This yields a standardised effect size of Cohen’s d=0.62 with an α error probability of 0.05 and desired power of 80% (1 – β=0.80). GPower indicates that 36 participants per group (72 total) are required to detect the specified effect size. To allow for up to 10% attrition, the total sample size was increased to 80 participants (40 per group).

Given that this is a multicentre trial conducted across four sites, we initially adjusted the sample size as per CONSORT recommendations for non-pharmacological treatments,^63^ to account for potential clustering effects due to differences between sites or instructors. The design effect was calculated by drawing on estimates from multicentre stroke rehabilitation literature, which typically report ICCs ranging from 0.007 to 0.031.^64^ An ICC of 0.01 was conservatively applied, as applying a higher ICC would have led to a larger sample size that was beyond the financial constraints of the current study and would have been unfeasible. Since the intervention will be delivered across four sites, we adjusted for potential clustering using a calculated design effect of 1.17 using the formula [1 + (*n*−1) × ICC], where *n* is the average cluster size and ICC is the intracluster correlation coefficient, which yielded a cluster-adjusted sample size requirement of 84 participants (approximately 21 per site).

### Quantitative data analysis

All quantitative data will be analysed using both descriptive and inferential statistical methods. Prior to conducting hypothesis testing, data will be assessed for completeness, normality and the presence of outliers. The distribution of continuous variables will be evaluated using visual inspection and the Shapiro-Wilk test to determine the suitability of parametric or non-parametric analyses. Baseline characteristics between the intervention and control groups will be compared using Mann-Whitney U tests for continuous variables and χ^2^ tests for nominal variables to assess group equivalence. All analyses will follow the intention-to-treat principle, meaning that all participants will be analysed in the groups to which they were originally randomised, regardless of adherence or dropout. Any missing data will be managed using appropriate imputation strategies, depending on the pattern and mechanism of missingness. The effects of the intervention on primary and secondary outcomes will be examined using mixed-model repeated measures ANOVA (analysis of variance) or appropriate non-parametric alternatives, with time (baseline, postintervention, 3-month follow-up) and group (RGM vs control) as factors.

To address multiplicity in the analysis of the 13 secondary outcomes (FES-I, Memory Test, Victoria Stroop Test, Rey Complex Figure Test, SPPB, 10MWT, 6MWT, ODTA, 9HPT, SIS-16, MADRS, RAND-36 and EQ-5D-5L), the Benjamini-Hochberg False Discovery Rate (FDR) procedure will be applied to control the expected proportion of false positives. Effect sizes of the variables will be expressed using partial eta squared. All statistical analyses will be conducted using IBM SPSS Statistics (V.29.0.1), with the FDR procedure implemented using the SPSS syntax. A significance level of p<0.05 will be applied for the primary outcome, while FDR-adjusted p values will be used for secondary outcomes.

### Qualitative data analysis

Qualitative data from focus group interviews will be transcribed verbatim from audio recordings into Swedish. The data will then be analysed using reflexive thematic analysis, as described by Braun and Clarke,^65^ with additional methodological guidance from Gale *et al*,^66^ supported by NVivo software to ensure systematic coding. The analysis will be conducted inductively, with themes derived directly from the data. The anonymised transcripts will be read repeatedly and independently by four researchers to identify meaning units and acquire a comprehensive understanding of the material. Meaning units, comprised of words or sentences corresponding to the research questions, will be compared until consensus is reached between researchers. These units will then be condensed into codes that remain close to the manifest content of the text, describing the material concisely while preserving its core meaning. The constant comparison method will be employed to synthesise data throughout the process. Subsequently, the codes will then be grouped into categories containing similar utterances under descriptive concepts. These categories will be merged into broader themes, each labelled with a description of their contents. Final themes will be agreed on by all members of the research team. Direct quotes from interviews will be included in the final synthesis to support findings and enhance credibility and trustworthiness.

## Ethics and dissemination

Ethical approval for this study has been obtained from the Swedish Ethical Review Authority (Etikprövningsmyndigheten, Dnr: 2025-01269-01). An amendment to expand the study from three to four centres was subsequently approved by the Swedish Ethical Review Authority (Dnr: 2025-08232-02). All participants will be fully insured during the course of the project, including travel to and from assessment and training locations, and during the training itself.

The current approved protocol version published on ClinicalTrial.gov registry (NCT06979050) is version 1 dated May 2025. Any modifications to the study protocol will be submitted as amendments to the Swedish Ethical Review Authority before implementation. Furthermore, during the course of the study, the participants will retain the right to withdraw from the study at any point without providing justification, and any data collected prior to withdrawal will be included in the final analysis according to the intention-to-treat principle.

Communication regarding the trial progress will be maintained through regular updates via professional networks of clinicians and RGM practitioners. On completion, trial findings will be submitted for publication in peer-reviewed rehabilitation and stroke journals, presented at national and international conferences focused on neurological rehabilitation and stroke recovery, and shared with relevant stakeholders and patient advocacy groups such as Strokeförbundet. The outcomes of this research will contribute to the evidence base for music-based interventions in stroke rehabilitation and inform the development of future clinical guidelines and research initiatives in this field.

## Supplementary material

10.1136/bmjopen-2025-107178 online supplemental file 1

10.1136/bmjopen-2025-107178 online supplemental file 2

10.1136/bmjopen-2025-107178 online supplemental file 3

10.1136/bmjopen-2025-107178 online supplemental file 4

10.1136/bmjopen-2025-107178 online supplemental file 5

10.1136/bmjopen-2025-107178 online supplemental file 6

10.1136/bmjopen-2025-107178 online supplemental file 7
