# Supplementary material for: Evaluation of the Ronnie Gardiner Method in individuals with stroke in the late phase of recovery: a protocol for a single-blind multicentre randomised controlled trial
Source: BMJ Open. 2026 Feb 4;16(2):e107178. doi: 10.1136/bmjopen-2025-107178 (PMC12878266; doi:10.1136/bmjopen-2025-107178)
Supplement: online supplemental file 2 [file bmjopen-16-2-s002.pdf]

Trial registeration information

Data category	Information
Primary registry and trial identifying number	ClinicalTrials.gov NCT06979050
Date of registration in primary registry	2025-05-18
Secondary identifying numbers	None declared
Source(s) of monetary or material support	Grants from: The Hjalmar Svenssons Foundation; Rune and Ulla Amlövs Foundation; Per-Olof Ahls Foundation; Tornspiran Foundation; Section for Neurology of the Swedish Association of Physiotherapists; Anna Ahrenberg Foundation; The Royal Swedish Academy of Music; Emil and Maria Palm Foundation; Wilhelm and Martina Lundgren Foundation; Ragnhild and Einar Lundström Memorial Fund
Primary sponsor	University of Gothenburg (Sahlgrenska Academy)
Secondary sponsor(s)	-
Contact for public queries	Petra Pohl, PhD, Department of Health and Rehabilitation, Institute of Neuroscience and Physiology, Sahlgrenska Academy, University of Gothenburg; Petra.pohl@neuro.gu.se
Contact for scientific queries	Petra Pohl, PhD; Petra.pohl@neuro.gu.se
Public title	Evaluation of the Ronnie Gardiner Method for balance improvement in chronic stroke survivors
Scientific title	Evaluation of the Ronnie Gardiner Method in Individuals with Stroke in Late Phase of Recovery: A Protocol for a Single-Blind Multicentre Randomized Controlled Trial
Countries of recruitment	Sweden (Gothenburg, Karlstad, Stockholm, Malmö)
Health condition(s) or problem(s) studied	Chronic stroke (≥ 6 months post-stroke)
Intervention(s)	Ronnie Gardiner Method group training (60 min, twice weekly for 12 weeks) vs. passive waitlist control
Ages eligible for study	≥ 18 years
Sexes eligible for study	All sexes

Data category	Information
Accepts healthy volunteers	No
Inclusion criteria	Community-dwelling stroke survivors ≥ 18 years; stroke > 6 months prior; MoCA ≥ 25; mRS 1–3; able to stand unassisted 2 min and walk 10 m (with/without device); independent travel to sites and restroom use
Exclusion criteria	Severe visual/hearing impairments; RGM training within past year (after Aug 2024); previous musical-instrument experience (> 1 h/week in past 10 years)
Study type	Interventional (randomized controlled trial)
Allocation	Randomized 1:1 (RGM vs. waitlist control)
Primary purpose	Rehabilitation
Phase	Not applicable
Date of first enrolment	Anticipated June 2025 (recruitment over an 2-year period)
Target sample size	84 participants (42 per group)
Recruitment status	Recruiting
Primary outcome(s)	Change in balance performance measured by Mini-BESTest
Key secondary outcomes	Gait (10-Meter Walk Test, 6-Minute Walk Test, Short Physical Performance Battery); upper limb (9-Hole Peg Test, Observational Drinking Task); cognition (Victoria Stroop Test, Rey Complex Figure Test, Memory Test); fear of falling (Falls Efficacy Scale-International); functional impact (Stroke Impact Scale-16); quality of life (RAND-36, EQ-5D-5L); depressive symptoms (MADRS)
