# Supplementary material for: Evaluation of the Ronnie Gardiner Method in individuals with stroke in the late phase of recovery: a protocol for a single-blind multicentre randomised controlled trial
Source: BMJ Open. 2026 Feb 4;16(2):e107178. doi: 10.1136/bmjopen-2025-107178 (PMC12878266; doi:10.1136/bmjopen-2025-107178)
Supplement: online supplemental file 3 [file bmjopen-16-2-s003.pdf]

Supplementary file 2

Interview guide for focus group interviews (English version)

For Participants

Overall areas:

Participation in RGM
Opening question: *How do you experience your participation in RGM?*

Life in General
Opening question: *How do you feel that your participation in RGM has affected your everyday life?*

- Can you tell us a little about what your daily life looks like today?
- Do you feel that your ability to be active (both socially and physically) has changed after your stroke?
- Do you feel that your participation in RGM in any way has affected your everyday life (in general)?
- RGM is an exercise performed together with others. Do you think this has affected your experience? In what way?

Follow-up questions will be asked to capture different time perspectives: how participants experience their participation **before**, **during**, and **after** the group sessions.

- **Before** may involve planning, prioritization, stress, and perceptions of opportunities/barriers to participation, as well as general aspects of daily life.
- **During** may explore how group dynamics affect well-being, whether there is competition among participants, comparison of performance, motivation, experiences of music, tempo, and exercise selection.
- **After** may involve how participation affects daily life more broadly, as well as perspectives on transferability to other situations and the ability to maintain the effects.

For Certified RGM Practitioners

Preparatory Work

- How have the preparations been: time investment, choice of music and exercises, what were the choices based on?

During the Sessions

- What is the interaction with the participants like, and what can be done to improve it? What is done to support group dynamics?
- Has the training needed to be adapted for the target group (stroke in the late phase)? If so, how – considering music choice, tempo, and exercises?
- Reflections on participant reactions (fatigue, boredom, joy, energy, etc.), both during exercises and at arrival/departure.
- Has any follow-up work been required? If so, in what way?

Swedish version

Intervjuguide för fokusgruppintervjuer (Swedish version)

Till deltagarna

Övergripande områden:

Deltagande i RGM
Ingångsfråga: *Hur upplever ni ert deltagande i RGM?*

Livet i stort
Ingångsfråga: *Hur upplever deltagare att deras deltagande i RGM har påverkat er vardag?*

- Kan ni berätta lite om hur er tillvaro ser ut idag?
- Upplever ni att era möjligheter att vara aktiv (såväl socialt som fysiskt) har förändrats efter er stroke?
- Upplever ni att ert deltagande i RGM på något vis har påverkat er vardag (i stort)?
- RGM är en träning som sker tillsammans med andra. Tycker ni att detta har påverkat er upplevelse? På vilket sätt?

Följdfrågor ställs för att fånga olika tidsperspektiv, dvs hur deltagare upplever sitt deltagande **inför**, **under** samt **efter**gruppträningen.

- **Inför** kan handla om behov av planering, prioritering, stress mm och fånga perspektiv på möjligheter/hinder för deltagande men även mer övergripande personens vardag.
- **Under** kan handla om hur gruppdynamiken påverkar måendet, finns tävlingsmoment mellan deltagarna (konkurrens), jämför man sig med de andras prestationer, blir man sporrad. Hur upplevs musik, tempo och val av övningar.
- **Efter** kan handla om hur deltagande påverkar personens vardag i ett vidare perspektiv men även fånga perspektiv på metodens överförbarhet till andra situationer, förmåga att upprätthålla mm.

Certifierade RGM-instruktörer

Förberedande arbetsinsatser

- Hur har förberedelserna sett ut: tidsåtgång, val av musik och övningar, vad baserades valen på?

Under träningen

- Hur är kontakten med deltagarna, vad kan göras för att förbättra den? Vad görs för gruppdynamiken i gruppen?
- Har träningen behövt anpassas till målgruppen stroke i sent skede? I så fall hur med tanke på val av musik, tempo och övningar?
- Reflektioner kring träningen avseende reaktioner hos deltagarna (trötthet, uttråkade, glada, pigga, etc) både vid själva övningarna och vid ankomst och hemgång.
- Har efterarbete fått göras? På vilket sätt?
