# Supplementary material for: Evaluation of the Ronnie Gardiner Method in individuals with stroke in the late phase of recovery: a protocol for a single-blind multicentre randomised controlled trial
Source: BMJ Open. 2026 Feb 4;16(2):e107178. doi: 10.1136/bmjopen-2025-107178 (PMC12878266; doi:10.1136/bmjopen-2025-107178)
Supplement: online supplemental file 4 [file bmjopen-16-2-s004.pdf]

RESEARCH PARTICIPANTS WITH STROKE WANTED

We are looking for people with stroke to evaluate the effects of the instructor-led rhythm- and music-based training method Ronnie Gardiner Method

During the fall, we will conduct a study in Gothenburg, Stockholm, Malmö, and Karlstad. The aim is to evaluate the effects on walking and balance ability, arm and hand function, and health-related quality of life in people who have had a stroke and who either receive joint group training using the Ronnie Gardiner Method twice a week for 12 weeks or delayed training using the same program after the final assessment in the study. At the end of the training period, group interviews will also be conducted to discuss how the training has affected everyday life.

Target group: Men and women, 18 years and older

Criteria for participation

- Your stroke must have occurred at least 6 months ago
- You must have some preserved function in the affected arm and hand
- You must be able to walk 10 meters independently
- You must be able to use the toilet independently
- You must not have severe hearing or vision impairment

The training, which is scheduled to start in September 2025, will be held two days a week, one hour per session, for 12 weeks in Gothenburg (Folkuniversitetet, Järntorget), Stockholm (Neurological Rehabilitation Clinic, Farsta), Malmö, and Karlstad (Resource Center). The training is free of charge, but you will need to make your own way to the relevant location. You will also be required to undergo assessments on three occasions.

Would you like to participate or receive more information?

Regardless of your place of study, please register with project manager Petra Pohl and her colleagues at the Department of Neuroscience and Physiology. You can register by email at petra.pohl@neuro.gu.se or by phone: XXXXXX.

The study has been approved by the Swedish Ethical Review Authority. Your participation is voluntary, and you may withdraw at any time. The information you provide will be processed in accordance with the General Data Protection Regulation (GDPR).
