# Supplementary material for: Evaluation of the Ronnie Gardiner Method in individuals with stroke in the late phase of recovery: a protocol for a single-blind multicentre randomised controlled trial
Source: BMJ Open. 2026 Feb 4;16(2):e107178. doi: 10.1136/bmjopen-2025-107178 (PMC12878266; doi:10.1136/bmjopen-2025-107178)
Supplement: online supplemental file 5 [file bmjopen-16-2-s005.pdf]

Information for Research Participants (English version)

Evaluation of the rhythm- and music-based training method Ronnie Gardiner Method for people with stroke

Information for research participants

We would like to ask if you would like to take part in a research project. This document provides information about the project and what participation entails.

What is this project and why do we want you to participate?

Stroke can cause long-term problems with walking and balance, and the risk of falling is often high. The Swedish rhythm- and music-based Ronnie Gardiner Method has the potential to positively affect walking ability and balance in an enjoyable and motivating way and is performed in a group setting. There is evidence that the Ronnie Gardiner Method is effective for people with Parkinson’s disease, but there is currently no scientific support for its effects after stroke. We are now looking for participants over 18 years of age, living independently, and who had a stroke more than 6 months ago.

The primary aim of the project is to evaluate the effects on walking and balance ability after 12 weeks of Ronnie Gardiner Method training, compared with a control group that maintains their usual lifestyle during the project period. Secondary aims include evaluating effects on cognitive function, hand function, well-being, and mood.

The project sponsor is the University of Gothenburg. The training will take place locally in Stockholm, Karlstad, Malmö, or Gothenburg. The project has been approved by the Swedish Ethical Review Authority (Ethics approval number: 2025-01269-01).

Time commitment and scheduling of assessments before the project starts and during follow-up

If you choose to participate, you will be invited to an initial visit where six physical tests will be conducted to assess your walking and balance abilities, as well as leg and arm function. You will also take cognitive tests (memory, spatial awareness, and processing speed) and complete five questionnaires regarding your well-being. The first visit takes about two hours and will be conducted locally in Stockholm, Karlstad, Malmö, or Gothenburg.

After these assessments, you will be randomly assigned to either a training group (participating in Ronnie Gardiner Method training sessions twice a week) or a control group (continuing with your usual lifestyle). If assigned to the control group, you will be offered the training after the third and final assessment.

After the 12-week training period, a follow-up assessment will be conducted using the same tests and questionnaires, and a final follow-up will take place three months later, for a total of three assessments. If you are in the training group, you will also

be invited to a group discussion with a researcher and other participants to share your experience of the training.

Possible consequences and risks of participating

It is well established that regular physical activity has positive health effects and reduces the risk of many lifestyle diseases. The Ronnie Gardiner Method is designed to improve walking and balance, and because the arms and legs move in rhythm with the music, it also provides beneficial exercise for the heart and lungs. If you have not exercised before, you may feel uncomfortable when your heart rate and breathing increase, but this is normal and usually subsides after a few minutes of rest. Previous studies have shown that the Ronnie Gardiner Method often has positive effects on mood and well-being.

Since both the tests and training challenge balance, there is a small risk of falling during assessments or training. To minimize this risk, assessments will be performed by experienced physiotherapists, and certain safety measures will be taken during training. If your balance is severely affected, training can be done seated until you feel comfortable standing. You can withdraw from the project at any time, and you are always welcome to contact the project leader to discuss any concerns or risks.

What happens with your data?

The project will collect and register information about you. The source of this information is you, your test results, and your questionnaire responses. In Stockholm, your participation will also be recorded in the medical journal, as the facility conducting the assessments (Stiftelsen Stora Sköndal) has an agreement with Region Stockholm requiring this by law.

Your data will be securely stored on protected computers at the University of Gothenburg and will be coded so that you cannot be identified. The key linking your code to your identity will be kept for 10 years for review purposes. Only the principal researcher Petra Pohl will have access to the data. Researchers at Karlstad University will only access pseudonymized data for group analysis after the final assessments.

Your responses and results will be protected from unauthorized access. Under the EU General Data Protection Regulation (GDPR), you have the right to access your data, correct any inaccuracies, request deletion of your data, or limit how it is used. However, deletion or limitation is not possible for data that is essential to the ongoing research.

You may contact the Data Protection Officer at dataskyddsombud@gu.se. If you are dissatisfied with how your personal data is handled, you can file a complaint with the Swedish Authority for Privacy Protection.

What happens to your test results?

All test results will be pseudonymized, meaning they cannot be directly linked to you. The key will be stored securely in the university archive, along with the original assessment documents.

You have the right to refuse permission to store your test results. If you give permission and later change your mind, your data will be destroyed or anonymized. If you wish to withdraw your consent, please contact Petra Pohl at the Department of Neuroscience and Physiology, Arvid Wallgrens backe, house 2, 413 46 Gothenburg, phone: +46 725 629882.

Your test results will only be used according to your consent. If you approve the use of your data for future research, this will require separate consent. If new research arises, the Ethics Authority will determine if you need to be contacted again.

How will you receive the project results?

When the project is complete, and the data has been analyzed, you may request a final report from the principal researcher. If you wish to receive your individual results, please contact Petra Pohl. You can also choose not to receive any results.

If any unexpected findings arise during the assessments or training, the principal researcher will contact you to discuss any necessary follow-up, such as medical care.

The project results will be published at group level in international scientific journals, at scientific conferences, and in the University of Gothenburg's research database (GUPEA).

Insurance and compensation

All participants are fully insured during the project, including travel to and from assessment and training locations, and during the training itself: in Karlstad through Resurscentrum (Karlstad Municipality), in Gothenburg through Folkuniversitetet, and in Stockholm through the Patient Injury Insurance (as the assessments take place at a facility with an agreement with Region Stockholm). Note: this insurance does not cover training performed independently at home.

Training with a licensed Ronnie Gardiner Method instructor is free of charge. No compensation is provided for lost work time or travel expenses. Participants in Stockholm are required to pay a patient fee, which will be reimbursed upon submission of receipts (instructions will be provided later).

Participation is voluntary

Participation is voluntary, and you can withdraw at any time without giving a reason. If you decide not to participate or to withdraw, it will not affect your future care or treatment.

If you wish to withdraw, please contact the project leader.

Evaluation of the rhythm and music-based training method Ronnie Gardiner Method for people who have had a stroke

Project leader

Petra Pohl, Senior Lecturer and Licensed Physiotherapist
Department of Neuroscience and Physiology
Arvid Wallgrens backe, house 2
413 46 Gothenburg
Phone: +46 31 7865737 or +46 725 629882
Email: petra.pohl@neuro.gu.se

Information till forskningspersoner (Swedish version)

Vi vill fråga dig om du vill delta i ett forskningsprojekt. I det här dokumentet får du information om projektet och om vad det innebär att delta.

Vad är det för ett projekt och varför vill vi att du ska delta?

Stroke kan ge långvariga problem med gång- och balansförmåga och fallrisken är ofta hög. Den svenska rytm- och musikbaserade Ronnie Gardiner Method har potential att påverka både gångförmågan och balansen under trevliga och lustfyllda former och utförs i grupp. Det finns belägg för att Ronnie Gardiner Method är verksamt för personer med Parkinsons sjukdom, men det saknas vetenskapligt stöd för effekterna efter stroke. Vi söker nu dig som är över 18 år, bor i eget boende och drabbades av stroke för mer än 6 månader sedan.
Syftet med projektet är primärt att utvärdera effekter på gång- och balansförmågan efter 12 veckors träning med Ronnie Gardiner Method, jämfört med en kontrollgrupp som lever som vanligt under projekttiden. Sekundärt utvärderas också effekter på tankemässig förmåga, handfunktion, välmående och sinnesstämning.
Forskningshuvudman för projektet är Göteborgs universitet. Med forskningshuvudman menas den organisation som är ansvarig för projektet. Träningen genomförs dock lokalt i Stockholm, Karlstad, Malmö eller Göteborg.
Forskningen är godkänd av Etikprövningsmyndigheten, diarienummer för prövningen hos Etikprövningsmyndigheten är 2025-01269-01.

Tidsåtgång och bokning av bedömning inför projektstart och uppföljning

Om du väljer att delta kommer du att kallas till ett första besök och genomföra sex fysiska test för att utvärdera din gång- och balansförmåga samt ben- och armfunktion. Du får också genomföra tankemässiga test (minne, rumslig uppfattning och tankesnabbhet). Slutligen får du svara på fem frågeformulär om ditt mående. Det första besöket tar ungefär två timmar i anspråk och sker lokalt i Stockholm, Karlstad, Malmö eller Göteborg.
Efter att de ovanstående mätningarna är utförda kommer du att slumpmässigt tilldelas en grupp där den ena gruppen erbjuds ledarledd träning med Ronnie Gardiner Method vid två träningstillfällen i veckan och den andra gruppen (kontrollgrupp) lever som vanligt under projektperioden (om du tilldelats kontrollgruppen kommer du att erbjudas samma träning när den tredje och sista mätningen genomförts).
Direkt efter träningsperiodens slut (efter tolv veckor) sker en uppföljande mätning av de ovan beskrivna testerna och frågeformulären, och slutligen sker en sista uppföljande mätning efter ytterligare tre månader, totalt sker alltså mätningar vid tre tillfällen. Om du tilldelats träningsgruppen kommer du även bjudas in till ett gruppsamtal tillsammans med en forskare och några andra gruppdeltagare för att diskutera hur ni har upplevt träningen.

Möjliga följder och risker med att delta i projektet

Det är sedan länge känt att regelbunden fysisk aktivitet och träning ger positiva hälsoeffekter och minskar risken för insjuknande i flertalet livsstilssjukdomar. Ronnie Gardiner Method är en träningsmetod som i första hand syftar till att påverka gång- och balansförmågan, men då kroppens armar och ben rörs i takt till musiken sker också gynnsam träning av lungor och hjärta. Om du aldrig har tränat tidigare kan det kännas obehagligt att hjärtats och andningens rytm ökar i takt, detta är dock

förväntat och går i regel över efter några minuters vila. Tidigare studier har visat att Ronnie Gardiner Method som genomförs i grupp med en inspirerande ledare ofta leder till positiva effekter vad gäller sinnesstämningen och välmående.
Eftersom både tester och träning utmanar balansen finns en viss risk att du råkar ut för en fallhändelse i samband med bedömning eller träning. För att minska risken sker bedömningarna av erfaren fysioterapeut, och i samband med träningen görs vissa åtgärder för att minska fallrisken. Om balansen är mycket påverkad avstås från att utmana balansen ytterligare och träningen kan då göras sittande tills du känner dig trygg att stå upp. Du får när som helst avbryta ditt deltagande och du är alltid välkommen att kontakta projektledaren under projektperioden om du önskar diskutera följder och risker med deltagandet.

Vad händer med dina uppgifter?

Projektet kommer att samla in och registrera information om dig. Källa till uppgifterna är du själv och de resultat på de fysiska och tankemässiga tester som du har utfört samt dina svar på frågeformulären. I Stockholm journalförs även din medverkan i projektet då enheten som genomför bedömningen och träningen (Stiftelsen Stora Sköndal) har avtal med Region Stockholm vilken kräver journalföring enligt lag. Informationen hanteras och förvaras på behörighetsskyddad dator på Göteborgs universitets datanätverk och enbart i form av koder där kodnyckeln förvaras i 10 år för att möjliggöra granskning. Ingen information kan härledas till dig som person. Endast huvudansvarig forskare Petra Pohl har tillgång till uppgifterna, dock kommer medverkande forskare vid Karlstad universitet få tillgång till kodade (psedonymiserade) data för databearbetning på gruppnivå efter sista mätningen.
Dina svar och dina resultat kommer således att behandlas så att inte obehöriga kan ta del av dem. Enligt EU:s dataskyddsförordning har du också rätt att kostnadsfritt ta del av de uppgifter om dig som hanteras i projektet och vid behov få eventuella fel rättade. Du kan också begära att uppgifter om dig raderas samt att behandlingen av dina uppgifter begränsas. Rätten till radering och begränsning av uppgifter gäller dock inte när uppgifterna är nödvändiga för den aktuella forskningen.

Dataskyddsombud nås på dataskyddsombud@gu.se. Om du är missnöjd med hur dina personuppgifter behandlas har du rätt att ge in klagomål till Integritetsskyddsmyndigheten, som är tillsynsmyndighet.

Vad händer med dina testresultat?

Alla testresultat kommer att kodas (pseudonymiseras) vilket innebär att de inte kan kopplas direkt till dig som person. Kodnyckel förvaras i brandsäkert skåp i Göteborgs universitets arkiv tillsammans med originaldokumenten från bedömningarna så att inte obehöriga kan ta del av dem.

Du har rätt att utan förklaring säga nej till att testresultaten sparas. Om du samtycker till att resultaten sparas har du rätt att senare och utan förklaring ta tillbaka (ångra) det samtycket. Dina testresultat kommer i så fall att kastas eller avidentifieras. Om du vill ångra ett samtycke ska du kontakta huvudansvarig forskare Petra Pohl, Institutionen för neurovetenskap och fysiologi, Arvid Wallgrens backe hus 2, 413 46 Göteborg, telefon 0725 629882.
Testresultaten får bara användas på det sätt som du har gett samtycke till. Om du godkänner att vi får bevara och använda dina resultat för framtida ändamål måste du

samtycka specifikt till detta. Tillkommer forskning som ännu inte är planerad, kommer Etikprövningsmyndigheten att besluta om du ska tillfrågas på nytt.

Hur får du information om resultatet av projektet?

När projektet är avslutat och data är sammanställda kommer du, om du så önskar, få en slutrapport av huvudansvarig forskare. Om du vill ta del av dina individuella uppgifter och testresultat ska du kontakta huvudansvarig forskare Petra Pohl på nedanstående kontaktuppgifter. Du kan också välja att avstå från att ta del av resultatet.
Om det i bedömnings- eller träningssituationer uppstår oförutsedda händelser eller oförutsedda fynd upptäcks av en medverkande person, kontaktas huvudansvarig forskare som då kontaktar dig för att diskutera eventuella åtgärder, såsom att uppsöka sjukvård.
Resultatet av projektet sammanställs på gruppnivå och kommer att presenteras i internationell vetenskaplig tidskrift, vid vetenskapliga kongresser samt i Göteborgs universitets forskningsdatabas GUPEA.

Försäkring och ersättning

Samtliga forskningspersoner, oavsett bostadsort, är under projektperioden helförsäkrade för resor till och från bedömnings- respektive träningslokaler och vid själva träningen: i Karlstad via Resurscentrum (Karlstad kommun), i Göteborg vid Folkuniversitetet, och i Stockholm via Patientskadeförsäkringen då bedömning/träning sker på enhet som har avtal med Region Stockholm. Observera att försäkringen inte gäller om träningen sker i det egna hemmet på eget initiativ. Träningen med licensierad RGM-ledare är kostnadsfri. Ingen ersättning utgår för utebliven arbetstid eller resor. För personer som tränar i Stockholm krävs patientavgift som dock ersätts av projektet efter inlämning av kvitton (instruktioner ges senare).

Deltagandet är frivilligt

Ditt deltagande är frivilligt och du kan när som helst välja att avbryta deltagandet. Om du väljer att inte delta eller vill avbryta ditt deltagande behöver du inte uppge varför, och det kommer inte heller att påverka din framtida vård eller behandling.

Om du vill avbryta ditt deltagande ska du kontakta den ansvariga för projektet (se nedan).

Ansvarig forskare för projektet

Petra Pohl, universitetslektor och leg. fysioterapeut
Institutionen för neurovetenskap och fysiologi
Arvid Wallgrens backe hus 2
413 46 Göteborg
Telefon 031-7865737 eller 0725 629882
E-postadress: petra.pohl@neuro.gu.se
