# Supplementary material for: Evaluation of the Ronnie Gardiner Method in individuals with stroke in the late phase of recovery: a protocol for a single-blind multicentre randomised controlled trial
Source: BMJ Open. 2026 Feb 4;16(2):e107178. doi: 10.1136/bmjopen-2025-107178 (PMC12878266; doi:10.1136/bmjopen-2025-107178)
Supplement: online supplemental file 6 [file bmjopen-16-2-s006.pdf]

Evaluation of the rhythm and music-based training method Ronnie Gardiner Method for people who have had a stroke

Consent to participate in the project (English version)

I have received verbal and written information about the study and have had the opportunity to ask questions. I will keep the written information.

- I consent to participate in the project "Evaluation of the rhythm- and music-based training method Ronnie Gardiner Method for people with stroke."
- I consent to my personal data and test results being stored and handled as described in the participant information.

Place and date	Signature
	Name in print

□ I do not wish to receive a final report on the research results after the conclusion of the study.

Evaluation of the rhythm and music-based training method Ronnie Gardiner Method for people who have had a stroke

Samtycke till att delta i projektet S

Jag har fått muntlig och skriftlig information om studien och har haft möjlighet att ställa frågor. Jag får behålla den skriftliga informationen.

- Jag samtycker till att delta i projektet «Utvärdering av den rytm- och musikbaserade träningsmetoden Ronnie Gardiner Method för personer med stroke»

- Jag samtycker till att mina personuppgifter och testresultat sparas och hanteras på det sätt som beskrivs i forskningspersonsinformationen.

Plats och datum	Underskrift
	Namnförtydligande

□ Jag önskar **inte** få en slutrapport om forskningsresultaten efter studiens avslut.
