# Supplementary material for: Evaluation of the Ronnie Gardiner Method in individuals with stroke in the late phase of recovery: a protocol for a single-blind multicentre randomised controlled trial
Source: BMJ Open. 2026 Feb 4;16(2):e107178. doi: 10.1136/bmjopen-2025-107178 (PMC12878266; doi:10.1136/bmjopen-2025-107178)
Supplement: online supplemental file 7 [file bmjopen-16-2-s007.pdf]

Data Management Plan (DMP)

Project: Evaluation of the music-based intervention Ronnie Gardiner Method for individuals with stroke

Information owner/Principal Investigator: Petra Pohl

Checklist for the data management plan

**Version and date [year-month-date]**	Version 1.0<br>2025-06-27<br>Registered by: Anna-Karin Norrman, University of Gothenburg

1. Overview		
**Project name**	Evaluation of the music-based intervention Ronnie Gardiner Method for individuals with stroke	
		Checklista
**1.1 Project description**	The aim of the project is to evaluate the effects of 12 weeks of training using the rhythm- and music-based rehabilitation method Ronnie Gardiner Method (RGM) in individuals in the chronic phase after stroke (> 6 months post-stroke), compared to a control group without training. The evaluation will focus on cognitive and physical function as well as quality of life. Additionally, focus group interviews (4–6 participants per group) are planned with both the participants who received the RGM training and the RGM instructors, to explore how the training was experienced from different perspectives.	Relevant to the project?<br>☒ Yes<br>☐ No<br>☐ Don't know
**1.2 Primary investigator/researcher (person, institution, or organization)**	Petra Pohl, PhD<br>ORCID ID: 0000-0002-3447-6547	Relevant to the project?<br>☒ Yes<br>☐ No<br>☐ Don't know
**1.3 Contributing researcher(s) and/or organization(s) and their roles**	**Principal Investigator/Contact Person:** Petra Pohl, Senior Lecturer at the Sahlgrenska Academy. Responsible for creating and maintaining the Data Management Plan (DMP), applying for research funding, recruiting study participants, analyzing primary data, and writing manuscripts.<br>**Co-Investigator:** Shashank Ghai, Associate Senior Lecturer at Karlstad University. Responsible for publishing the study protocol, assessing participants post-intervention, analyzing post-intervention primary data, and writing manuscripts.	Relevant to the project?<br>☒ Yes<br>☐ No<br>☐ Don't know

	**Co-Investigator:** Charlotte Wassenius, Adjunct Senior Lecturer at the Sahlgrenska Academy. Conducts focus group interviews, analyzes qualitative data, and writes manuscripts.<br>**Co-Investigator:** Iolanda Santos Tavares, Adjunct Senior Lecturer at the Sahlgrenska Academy. Conducts focus group interviews, analyzes qualitative data, and writes manuscripts.<br>**Research Staff (Training Leaders):** Carina Svahn, Karin Eriksson Jeanson, Ingela Marklund, and Ulrika Bergström. Meet with study participants; do not analyze data.<br>**Expert Senior Researchers (do not analyze data):**<br>Professor Emeritus Christian Blomstrand<br>Senior Lecturer Emmelie Barenfeldt<br>Associate Professor Lina Bunketorp Käll	
**1.4 Research Principal**	University of Gothenburg	Relevant to the project?<br>☒ Yes<br>☐ No<br>☐ Don't know
**1.5 Responsible department/unit**	Institution of neuroscience and physiology, Dept of health and rehabilitation, Unit of physiotherapy.	Relevant to the project?<br>☒ Yes<br>☐ No<br>☐ Don't know
**1.6 Funding**	Funder and (if applicable) registration number:<br>Physiotherapy (unit), section of neurology<br>Anna Ahrenbergs Foundation<br>Wilhelm and Martina Lundgren Foundation, 2025-SA-4965<br>Emil and Maria Palm Foundation<br>Hjalmar Svensson Foundation, HJSV2025003<br>Royal Musical Academy<br>Ragnhild and Einar Lundström Foundation, LA2025-0072<br>Ulla and Rune Amlöv Foundation, 2025-485<br>Per-Olof Ahl Foundation, 2025-124<br>Tornspiran Foundation, 1067	Relevant to the project?<br>☒ Yes<br>☐ No<br>☐ Don't know
**1.7 Guidelines**	The guidelines followed are the University of Gothenburg's governing document *Rules for Research Data* (Ref. No. GU 2025/422), as well as the *Guidelines for Information Classification*, the *IT Security Policy*, and the procedures for creating a Data Management Plan (DMP). Some funders require that grants be used within a specified time frame, and all require written reporting.	Relevant to the project?<br>☒ Yes<br>☐ No<br>☐ Don't know

2. Protect the research data

**2.1 Information security and classification**	According to the governing document *Guidelines for Information Classification*, the data in this project are classified as Level 3, as health data will be collected using self-report questionnaires. This requires that digital data be stored using Secure Storage in accordance with the University of Gothenburg's procedures.<br><br>Paper-based assessment instruments will be archived in the local archive of the unit, together with the code key. Only the project leader and the administrator have access to the archive.	Relevant to the project?<br>☒ Yes<br>☐ No<br>☐ Don't know
**2.2 Ethical review**	Ethical approval was obtained on March 21, 2025.<br><br>Case number: 2025-01269-01.	Relevant to the project?<br>☒ Yes<br>☐ No<br>☐ Don't know
**2.3 Confidential information**	The data include certain personal information (name, address, telephone number, and email address). No personal identity numbers will be recorded.	Relevant to the project?<br>☒ Yes<br>☐ No<br>☐ Don't know
**2.4 Information about personal data processing**	Yes, personal data will be processed. Study participants will receive both written and oral participant information (approved by the Ethical Review Authority) and will be asked to provide written informed consent.	Relevant to the project?<br>☒ Yes<br>☐ No<br>☐ Don't know
**2.5 Protection of participant identity**	Data containing personal information will be stored according to the Secure Storage procedures and handled on the TRE platform, in accordance with the University of Gothenburg's recommendations. Data will be pseudonymized with a code as soon as inclusion occurs.	Relevant to the project?<br>☒ Yes<br>☐ No<br>☐ Don't know
**2.6 Data protection officer**	The University of Gothenburg's "Checklist for Processing Personal Data" has been followed according to the instructions. Step 1 is to conduct an information classification, step 2 to perform a threshold analysis, step 3 to create a Data Management Plan (DMP), step 4 to apply for ethical approval, step 5 to conduct a data protection impact assessment if applicable, step 6 to enter into agreements	Relevant to the project?<br>☒ Yes<br>☐ No<br>☐ Don't know

	if applicable, step 7 to obtain consent, and step 8 to report the processing of personal data. All steps have been completed.	
**2.7 Intellectual property rights/Copyright**	The project uses materials that are copyrighted by the Ronnie Gardiner Method Fundraising Foundation (RGMFF). Permission to use the materials has been obtained from the Foundation.	Relevant to the project?<br>☒ Yes<br>☐ No<br>☐ Don't know
**2.8 Agreements with other parties**	An agreement will be drafted with co-investigator Shashank Ghai at Karlstad University, as he will analyze data via the shared TRE platform. A legal advisor will be contacted shortly.	Relevant to the project?<br>☒ Yes<br>☐ No<br>☐ Don't know
**2.9 Limitations to access**	Access rules will likely not be necessary.	Relevant to the project?<br>☐ Yes<br>☒ No<br>☐ Don't know
**2.10 Embargo**	Not applicable in the planned study.	Relevant to the project?<br>☐ Yes<br>☒ No<br>☐ Don't know

3. Collect or produce the research data

**3.1 Type of data**	Quantitative data in the form of cognitive and physical measurements as well as self-report questionnaires will be collected at three time points for 84 individuals. In addition, qualitative data will be collected as audio recordings of focus group discussions led by a moderator at one occasion (though with several groups), which will subsequently be transcribed verbatim.	Relevant to the project?<br>☒ Yes<br>☐ No<br>☐ Don't know
**3.2 Existing data**	No existing data from previous studies are available for reuse.	Relevant to the project?<br>☐ Yes<br>☒ No<br>☐ Don't know

**3.3 Data collection**	Quantitative data**:** Multiple measurement instruments will be used for the cognitive and physical assessments (e.g., Mini-BESTest, MoCA, 10-meter walk test, 6-minute walk test, among others). Five self-report questionnaires will also be used (e.g., MADRS, RAND-36, and EQ-5D). All data collection will take place at three time points: baseline, post-intervention, and at a 3-month follow-up. The measurements will be conducted by the project investigator Petra Pohl (baseline and post-intervention), co-investigator Shashank Ghai (3-month follow-up), and physiotherapists Ingela Berglind and Ulrika Björkman (post-intervention and 3-month follow-up). For the measurements, paper copies of the instruments, pens, erasers, stopwatches, a soft cushion, and a shoebox are needed.<br><br>Focus group interviews will be conducted post-intervention by two co-investigators (Charlotte Wassenius and Iolanda Santos). A voice recorder will be required for data collection. The audio files will later be transcribed verbatim (possibly in TRE).<br><br>All data will be collected in Stockholm, Karlstad, Malmö and Gothenburg.	Relevant to the project?<br>☒ Yes<br>☐ No<br>☐ Don't know

4. Document the research data

**4.1 Documentation**	A research diary has been kept since September 2023 and includes most of the decisions made. Multiple versions of the project plan exist, as well as applications to various potential funders. The ethics application with its accompanying appendices. Test protocols specifying the order of the tests. A description of how the "Drinking from a Glass" test was developed (a cheat sheet).<br><br>All documents are saved on the local server of the work computer in the main folder: Research GU → RGM RCT, with various subfolders such as Applications_Decisions; Participants; Measurements; Ethics Application, etc.	Relevant to the project?<br>☒ Yes<br>☐ No<br>☐ Don't know
**4.2 Metadata**	Metadata will be produced shortly. The ambition is to make them machine-readable. Guidance for formulation is available via the Swedish National Data Service (SND).	Relevant to the project?<br>☒ Yes<br>☐ No<br>☐ Don't know
**4.3 Terminology, ontologies, standards, controlled vocabularies etc.**	Not applicable.	Relevant to the project?<br>☐ Yes<br>☒ No<br>☐ Don't know

5. Organize the research data

**5.1 Folder structure**	Data will be stored according to the guidelines and recommendations within the framework of Secure Storage. The exact folder structure is not specified here at this time.	Relevant to the project?<br>☒ Yes<br>☐ No<br>☐ Don't know
**5.2 File formats**	SPSS files (.sav and .spv). Word format (.docx) for transcriptions used in NVivo (.nvp) for analysis, as well as for manuscript writing.<br>All file formats are provided by the University of Gothenburg, with associated IT support in case of system failure.	Relevant to the project?<br>☒ Yes<br>☐ No<br>☐ Don't know
**5.3 File naming convention**	Original files ("master files") will be named with "Original," the date, and the relevant time point, e.g., "baseline." Copies created from the original files can be named according to the content to be analyzed and the date.	Relevant to the project?<br>☒ Yes<br>☐ No<br>☐ Don't know
**5.4 Versioning**	As soon as new versions of files are created, they will be dated in the file name using the format year-month-day. If multiple versions are created on the same day, it may be necessary to add "version 1.x." The project investigator, Petra Pohl, is responsible for maintaining the master files and ensuring that internal rules for new versions are followed.	Relevant to the project?<br>☒ Yes<br>☐ No<br>☐ Don't know
**5.5 Storage and backup**	Data are stored in Secure Storage, the platform recommended by the University of Gothenburg. The Secure Storage environment is designed to handle and store data requiring strong protection against accidental or unauthorized access (including protection against advanced attacks intended to access the information). The service includes regular backups of stored data. Backups are retained for two weeks. Data restoration can be performed upon request by the data owner (the project leader) through IT support.	Relevant to the project?<br>☒ Yes<br>☐ No<br>☐ Don't know

6. Budget for managing the research data

**6.1 Staff**	Estimated costs for staff involved in data management:<br>- Data preparation and documentation: SEK 100,00<br>- Data collection: SEK 150,000 (quantitative and qualitative)<br>- Data processing: SEK 100,000 (qualitative)	Relevant to the project?<br>☒ Yes<br>☐ No<br>☐ Don't know

**6.2 Storage**	Certain costs are associated with the use of the Trusted Research Environment (TRE) sharing platform, although the exact amount is currently unclear.	Relevant to the project?<br>☒ Yes<br>☐ No<br>☐ Don't know
**6.3 Hardware and software**	Not applicable, as all software is provided by the University of Gothenburg.	Relevant to the project?<br>☐ Yes<br>☒ No<br>☐ Don't know

7. Preserve and make the research data accessible

**7.1 Prepare data and documentation**	Data will be prepared and made available in accordance with the University of Gothenburg's regulations, in consultation with the Research Data Support unit. The recommendation is that data containing personal information may be made available via the SND repository with so-called "restricted access." This means that a description of the data (metadata) will be published along with any documentation files, while the actual data files will not be openly accessible. Anyone wishing to access the data must submit a request, and each request will be reviewed before access is granted.	Relevant to the project?<br>☒ Yes<br>☐ No<br>☐ Don't know
**7.2 Metadata and keywords**	Metadata will be produced shortly.<br>Keywords: Physiotherapy; music-based intervention; Ronnie Gardiner Method; stroke rehabilitation	Relevant to the project?<br>☒ Yes<br>☐ No<br>☐ Don't know
**7.3 Preserve and make the data accessible**	The Principal Investigator is responsible for the preservation, archiving, and disposal of data, in consultation with the Head of Section. Paper-based measurements (raw data) will be archived at the Unit for Physiotherapy, Institute of Neuroscience and Physiology, Arvid Wallgrens Backe, Building 2. Digital data (raw data files) will be preserved in Secure Storage and TRE. The University of Gothenburg's *Rules for Research Data* will be strictly followed.<br>All measurement data from study participants will be published and made available, along with selected background information (e.g., age, sex, side of stroke), once all measurements and analyses are completed. Due to confidentiality reasons, interview recordings cannot be	Relevant to the project?<br>☒ Yes<br>☐ No<br>☐ Don't know

	published, although Swedish transcripts may be made available.<br>The SND repository is the recommended platform for data publication.<br>Planned time of publication: after the final publication, estimated in 2028–2029.	
**7.4 Responsible organization**	University of Gothenburg.	Relevant to the project?<br>☒ Yes<br>☐ No<br>☐ Don't know
**7.5 Limitations due to guidelines or legal/ethical restrictions**	Quantitative data: The full dataset will be made available on request.<br>Qualitative data: Interview files in which individual partici-pants may be identifiable will not be made available. Only the transcription files, in Swedish, will be shared.	Relevant to the project?<br>☒ Yes<br>☐ No<br>☐ Don't know
**7.6 Limitations due to hardware and software**	No specific software or tools are required to use the dataset. Files in SPSS and NVivo will be available in other formats, this will be done in consultation with the Research data support.	Relevant to the project?<br>☐ Yes<br>☒ No<br>☐ Don't know
**7.7 Data access rules and/or licenses**	The ambition is for the data (upon access request) to be reusable by others, as the Ronnie Gardiner Method (RGM) is still a relatively under-researched approach. However, this is subject to the limitation that the data contain perso-nal and sensitive information. Details regarding data hand-ling will be determined at a later stage.	Relevant to the project?<br>☒ Yes<br>☐ No<br>☐ Don't know
**7.8 Local research data support unit**	Once the quantitative data have been processed and the manuscript is ready for publication, the data will be made available by the Principal Investigator in consultation with the local research data support unit.	Relevant to the project?<br>☒ Yes<br>☐ No<br>☐ Don't know
**7.9 Certified data repository**	The recommended repository is the Svensk Nationell Data-tjänst (Swedish National Data Service, SND) repository. Contact will be made at a later stage. Guidance is also available through the University of Gothenburg's Research Support.	Relevant to the project?<br>☒ Yes<br>☐ No<br>☐ Don't know

**7.10 Persistent identifier** **(PID)**	Upon publication via the SND repository, the data will be assigned a persistent identifier in the form of a DOI (Digital Object Identifier).	Relevant to the project? ☒ Yes ☐ No ☐ Don’t know
