# Supplementary material for: Evaluation of the Ronnie Gardiner Method in individuals with stroke in the late phase of recovery: a protocol for a single-blind multicentre randomised controlled trial
Source: BMJ Open. 2026 Feb 4;16(2):e107178. doi: 10.1136/bmjopen-2025-107178 (PMC12878266; doi:10.1136/bmjopen-2025-107178)
Supplement: Supplementary file 8 [file bmjopen-2025-107178.reviewer_comments.pdf]

PEER REVIEW HISTORY

BMJ Open publishes all reviews undertaken for accepted manuscripts. Reviewers are asked to complete a checklist review form and are provided with free text boxes to elaborate on their assessment. These free text comments are reproduced below.

ARTICLE DETAILS

Title (Provisional)

Evaluation of the Ronnie Gardiner Method in Individuals with Stroke in Late Phase of Recovery: A Protocol for a Single-Blind Multicentre Randomized Controlled Trial

Authors

Ghai, Shashank; Pohl, Petra

VERSION 1 - REVIEW

**Reviewer**	**1**
**Name**	**Piemonte, Maria Elisa**
**Affiliation**	**Universidade de Sao Paulo Faculdade de Medicina, Physical Therapy**
**Date**	**09-Sep-2025**
**COI**	**None**

Overall, the protocol is clearly presented and addresses an important and clinically relevant research question. The proposed trial is well designed and has the potential to generate valuable evidence regarding the use of rhythm- and music-based interventions in stroke rehabilitation. Nevertheless, the manuscript could be further strengthened by revising and clarifying several methodological aspects. Addressing these points would enhance the transparency, reproducibility, and overall robustness of the study.

1- The sample size calculation, as currently presented, does not account for the multicentre design of the trial. Since the intervention will be delivered across three centres, clustering effects are likely (e.g., due to differences between sites or instructors), which can reduce the effective sample size and lead to underpowered analyses. According to CONSORT-NPT recommendations, trials of non-pharmacologic interventions should adjust their sample size for potential clustering by including an ICC and applying a design effect. To strengthen the protocol, we recommend revising the sample size section to explicitly model clustering.

2- The protocol includes a wide range of secondary outcomes spanning motor, cognitive, psychosocial, and quality of life domains. While this breadth is valuable, it raises concerns about multiplicity and the increased risk of type I error. To address this, we recommend that

the authors specify a hierarchy of outcomes or adopt a formal approach to control for multiplicity.

3- To enhance transparency and reproducibility, it would be highly beneficial to include a detailed table outlining the content of each intervention session. Such a table should describe the specific activities performed, the targeted functional domains, and the criteria for progression.

4- The qualitative component would benefit from a more detailed description of how data will be recorded, managed, and analyzed.

VERSION 1 - AUTHOR RESPONSE

Reviewer 1

Reviewer 1: Overall, the protocol is clearly presented and addresses an important and clinically relevant research question. The proposed trial is well designed and has the potential to generate valuable evidence regarding the use of rhythm- and music-based interventions in stroke rehabilitation. Nevertheless, the manuscript could be further strengthened by revising and clarifying several methodological aspects. Addressing these points would enhance the transparency, reproducibility, and overall robustness of the study.

Response: We sincerely thank the reviewer for the positive evaluation of our protocol and for recognizing the clinical relevance of our study. We are grateful for your constructive feedback, which has helped us to further strengthen the methodological clarity and transparency of our manuscript. We have carefully addressed each of the points raised, and the corresponding revisions have been made in the manuscript. Below, we provide a point-by-point response, indicating the specific changes along with the relevant page and line numbers.

Reviewer 1: 1- The sample size calculation, as currently presented, does not account for the multicentre design of the trial. Since the intervention will be delivered across three centres, clustering effects are likely (e.g., due to differences between sites or instructors), which can reduce the effective sample size and lead to underpowered analyses. According to CONSORT-NPT recommendations, trials of non-pharmacologic interventions should adjust their sample size for potential clustering by including an ICC and applying a design effect. To strengthen the protocol, we recommend revising the sample size section to explicitly model clustering.

Response: We sincerely thank the reviewer for this important observation. We acknowledge that in our initial evaluation, the sample size calculation did not adequately account for the multicenter design, which was indeed a shortcoming on our part. Following your expert recommendation, we have revised the power analysis to include a design effect adjustment that appropriately addresses potential clustering effects.

To accomplish this, we employed an ICC of 0.01, which falls within the range recommended in stroke rehabilitation literature (0.007 to 0.031). This conservative ICC estimate was selected following a comprehensive sensitivity analysis. We found that applying a higher ICC would result in a substantially larger sample size that would exceed our financial resources and prove unfeasible. Given our practical limitations, including restricted financial resources, limited availability of qualified trainers, and institutional capacity constraints, our maximum feasible sample size is 80 to 85 participants. We cannot realistically expand beyond this threshold given these resource limitations.

To address the clustering adjustment while maintaining feasibility, we strategically expanded our study from three to four sites. This expansion was necessary because with only three sites, the cluster-adjusted sample size requirement would have been 92 participants, which would not be feasible to implement across three sites alone. Therefore, we added a fourth site in Malmö, in addition to our original sites in Karlstad, Stockholm, and Gothenburg. This expansion allows us to distribute participants across additional centers while maintaining a realistic recruitment target of approximately 21 participants per site.

Using this four-site configuration, we calculated a design effect of 1.15, resulting in a cluster-adjusted sample size requirement of 84 participants across the four sites. This approach ensures that our study maintains adequate statistical power while remaining practically achievable within our resource constraints. The changes in the manuscript are mentioned at the following instance:

Page 13, Line 529-551: The sample size estimation was conducted using GPower software (version 3.1.9.7). The study's primary outcome is the between-group difference in change of Mini-BESTest total scores post-intervention. Based on Beauchamp, Niebuhr (61), a minimal clinically important difference of 4 points was chosen, with a corresponding baseline standard deviation (SD) of 6.5 points for the Mini-BESTest in a stroke outpatient cohort. This yields a standardized effect size of Cohen's d = 0.62 with an α error probability of 0.05 and desired power of 80% ($1 – \beta = 0.80$). GPower indicates that 36 participants per group (72 total) are required to detect the specified effect size. To allow for up to 10% attrition, the total sample size was increased to 80 participants (40 per group).

Given that this is a multicenter trial conducted across four sites, we initially adjusted the sample size as per CONSORT recommendations for non-pharmacological treatments (62), to account for potential clustering effects due to differences between sites or instructors. The design effect was calculated by drawing on estimates from multicenter stroke rehabilitation literature, which typically report ICCs ranging from 0.007 to 0.031 (63). An ICC of 0.01 was conservatively applied, as applying a higher ICC would have led to a larger sample size that was beyond the financial constraints of the current study and would have been unfeasible. Since the intervention will be delivered across four sites, we adjusted for potential clustering using a calculated design effect of 1.17 using the formula $[1 + (n-1) \times ICC]$, where n is the average cluster size and ICC is the intracluster correlation coefficient, which yielded a cluster-adjusted sample size requirement of 84 participants (approximately 21 per site).

Reviewer 1: 2- The protocol includes a wide range of secondary outcomes spanning motor, cognitive, psychosocial, and quality of life domains. While this breadth is valuable, it raises concerns about multiplicity and the increased risk of type I error. To address this, we recommend that the authors specify a hierarchy of outcomes or adopt a formal approach to control for multiplicity.

Response: We sincerely thank you for your thoughtful and valuable feedback regarding the potential for multiplicity in our study due to the wide range of secondary outcomes. We greatly appreciate your recognition of the value in including a broad set of outcomes spanning motor, cognitive, psychosocial, and quality-of-life domains, as this breadth is essential for comprehensively understanding the effectiveness of a complex healthcare intervention such as ours. We fully understand your concerns about the increased risk of type I errors associated with multiple outcome assessments. To address this, we have now included the Benjamini-Hochberg False Discovery Rate (FDR) procedure in our statistical analysis plan to control the expected proportion of false positives at a level of 0.05 for the 13 secondary outcomes. This formal approach ensures robust and reliable findings while preserving the ability to explore the multifaceted impacts of the intervention. The changes in the manuscript are mentioned at the following instance:

Page 14, Line 575-583: To address multiplicity in the analysis of the 13 secondary outcomes (FES-I, Memory Test, Victoria Stroop Test, Rey Complex Figure Test, SPPB, 10MWT, 6MWT, ODTA, 9HPT, SIS-16, MADRS, RAND-36, and EQ-5D-5L), the Benjamini-Hochberg False Discovery Rate (FDR) procedure will be applied to control the expected proportion of false positives. Effect sizes, of the variables will be expressed using partial eta squared. All statistical analyses will be conducted using IBM SPSS Statistics (Version 29.0.1), with the FDR procedure implemented using the SPSS syntax. A significance level of $p < 0.05$ will be applied for the primary outcome, while FDR-adjusted p-values will be used for secondary outcomes.

Reviewer 1: 3- To enhance transparency and reproducibility, it would be highly beneficial to include a detailed table outlining the content of each intervention session. Such a table should describe the specific activities performed, the targeted functional domains, and the criteria for progression.

Response: We thank the reviewer for this insightful comment and fully appreciate the importance of transparency and reproducibility. However, we would like to clarify that RGM is fundamentally a personalized, instructor-led intervention, and in our study the inclusion criteria involves recruiting participants with stroke who have modified Rankin Scale scores of 1-3, which represents a very wide range of functional abilities and diverse physical capabilities. Given the group-based nature of this intervention, sessions must be dynamically adapted in real-time to ensure activities are appropriately challenging and accessible for all participants simultaneously. For example, within the same group, participants with higher physical capacities can perform activities in standing positions while those with lower abilities have the option to perform identical activities seated, and the instructor must continuously assess and modify the tempo and complexity based on collective group performance. While all instructors, regardless of their professional background, receive standardized training in core intervention elements (including the consistent use of 19 symbols, structured warm-ups, and the systematic progression framework

detailed in our manuscript), the music selection is individualized when possible to incorporate participants' preferences, with tempo measured in beats per minute starting at 50 BPM and progressing according to group capabilities. We have also mentioned in the manuscript that a typical RGM session follows a consistent 60-minute structural framework beginning with five-minute warm-ups incorporating stretching and breathing exercises, and concludes with relaxation and social interaction periods.

Nonetheless, we strongly believe that creating a rigid session-by-session table would not accurately represent the responsive, individualized nature of this intervention and could potentially compromise its effectiveness if replicated without appropriate real-time adaptations. To address transparency concerns while maintaining intervention fidelity, we propose to include in our final results publication comprehensive details following the Robb et al. (2011) checklist for reporting music-based interventions as recommended by CONSORT guidelines. This will include detailed information on trainer qualifications and experience levels, group size, specific music selections with tempo progressions, and intervention strategy including the specific choreographed scores used from the 19 RGM symbols. We have also addressed these aspects in the manuscript, and the corresponding changes are detailed at:

Page 5-6, Line 214-272: The Ronnie Gardiner Method is a comprehensive neuroplasticity-based intervention that combines multisensory stimulation through rhythm, music, color, speech, text, shapes, and coordinated movements to enhance cognitive and motor functioning in individuals with neurological conditions. This evidence-based program is implemented across Swedish rehabilitation and healthcare centres and will be adapted for the current study population.

The intervention will utilize a unique symbol system comprising four distinctive red and blue symbols resembling hands and feet that will be projected on a screen within specialized note systems called “choreoscores” (41). The color-coding will represent lateralized brain activity, with red symbols corresponding to left hemispheric control of right-sided body movements and blue symbols representing right hemispheric control of left-sided movements. This system creates 19 possible symbol combinations; each associated with specific movements and four-letter verbal cues imitating sounds inspired from drumming (such as "BOOM", "CHIC", "TING") that participants will pronounce loudly while executing corresponding motor actions to rhythmical music. The multi-tasking nature of RGM will require participants to simultaneously process visual symbols, execute corresponding movements, vocalize specific verbal cues, maintain balance, follow musical rhythm, and attend to practitioner instructions. This complexity is designed to improve dual-task performance and movement automaticity while promoting neuroplastic adaptations through repeated practice of cognitively demanding motor tasks.

The RGM protocol will incorporate multiple external cueing modalities including visual cues (screen-projected symbols), auditory cues (beat-based popular music), and somatosensory cues (body percussion through handclaps, foot stamping, and thigh slapping). Music selection will be individualized, when possible, to incorporate participants' preferences, with tempo measured in beats per minute to enable objective progression monitoring. The intervention will be delivered in standing position to enhance postural stability through weight shifts that activate anticipatory

and reactive postural control mechanisms, with seated adaptations available, when necessary, based on individual participant needs.

Training sessions will be led by a certified RGM practitioner. Each 60-minute group session will follow a structured format beginning with a five-minute warm-up period incorporating light stretching, relaxation techniques, and breathing exercises. This will be followed by systematic progression through the symbol-based activities starting at a slow tempo of 50 beats per minute and gradually increasing in complexity and pace according to individual participant capabilities. Sessions will conclude with a five-minute relaxation period and opportunities for social interaction among participants.

The complete intervention protocol will involve twice-weekly group sessions conducted over 12 consecutive weeks under the guidance of the certified RGM practitioner who will adjust activity complexity based on participants' physical capacities. To ensure transparency and reproducibility, comprehensive implementation details will be reported in the final publication following the Robb, Story (42) checklist for reporting music-based interventions as recommended by CONSORT guidelines. This will include detailed information on trainer qualifications and experience levels, group size, specific music selections with tempo progressions, music delivery method, intervention strategies including the specific choreographed scores used from the 19 RGM symbols, intervention setting, and treatment fidelity.

Reviewer 1: 4- The qualitative component would benefit from a more detailed description of how data will be recorded, managed, and analyzed.

Response: We sincerely thank the reviewer for this important observation regarding the qualitative component. We have substantially revised the qualitative section to provide comprehensive details about data recording, management, and analysis procedures. The revised methodology now includes specific data recording and storage protocols that reference Swedish data protection regulations and Gothenburg University's data management guidelines. Moreover, we have now provided a detailed step-by-step analytical approach using established qualitative content analysis methods, and clear description of how rigor will be maintained through systematic meaning unit identification, and constant comparison methods. We have also mentioned how we intend to incorporate transparency measures including the use of direct quotes to support findings. We believe these additions address the reviewer's concerns about methodological transparency and provide sufficient detail for replication and evaluation of the qualitative component of our study. The changes are mention at:

Page 14, Line 585-604: Qualitative data analysis

Qualitative data from focus group interviews will be transcribed verbatim from audio recordings into Swedish. The data will then be analysed using reflexive thematic analysis, as described by Braun and Clarke [64], with additional methodological guidance from Gale, Heath [65], supported by NVivo software to ensure systematic coding. The analysis will be conducted inductively, with themes derived directly from

the data. The anonymized transcripts will be read repeatedly and independently by four researchers to identify meaning units and acquire a comprehensive understanding of the material. Meaning units, comprised of words or sentences corresponding to the research questions, will be compared until consensus is reached between researchers. These units will then be condensed into codes that remain close to the manifest content of the text, describing the material concisely while preserving its core meaning. The constant comparison method will be employed to synthesise data throughout the process. Subsequently, the codes will then be grouped into categories containing similar utterances under descriptive concepts. These categories will be merged into broader themes, each labelled with a description of their contents. Final themes will be agreed upon by all members of the research team. Direct quotes from interviews will be included in the final synthesis to support findings and enhance credibility and trustworthiness.
